# Statin treatment in routine clinical practice: Insights from the STATRIP physician survey

**DOI:** 10.1016/j.athplu.2026.01.003

**Published:** 2026-01-17

**Authors:** Panagiota Anyfanti, Christina Antza, Dimitrios Poulis, Konstantinos Konstantaros, Makrina Savvoulidou, Georgios Styliadis, Vasilios Kotsis

**Affiliations:** a Third Department of Internal Medicine, Papageorgiou Hospital, Aristotle University of Thessaloniki, Greece; b Medical School, Aristotle University of Thessaloniki, Greece

**Keywords:** Statins, Dyslipidemia, Survey, Knowledge, Physicians, Questionnaire

## Abstract

**Background:**

Statins remain to date the primary therapeutic option for dyslipidemia. However, a significant portion of patients with dyslipidemia fail to achieve optimal low-density lipoprotein targets for reasons often related to treating physicians. The aim of STAtin Treatment in Routine clinical Practice (STATRIP) survey was to report and quantify perceptions and common beliefs regarding treatment with statins, among physicians implicated in the primary and secondary care of patients with dyslipidemia.

**Methods and results:**

This observational cross-sectional study was conducted using an online-distributed questionnaire, which was designed to cover a wide range of physicians’ knowledge and perceptions on treatment with statins. A total of 261 health care providers filled out the survey, mostly general practitioners and internists (93.5 %). Study participants clearly expressed their concerns regarding statin-related side effects, including fears on interactions with other medication, muscle aches and pain, increase in liver enzymes, and gastrointestinal disorders. Myalgias were observed by physicians in as many as 29.2 % of patients receiving rosuvastatin, and in as many as 26.5 % receiving atorvastatin. Combination lipid-lowering therapy with ezetimibe was reported by only 53.6 % of participants as a prevalent strategy for uncontrolled individuals. Only 58.6 % apply non-HDL cholesterol measurements in their clinical practice.

**Conclusions:**

Our study provides a clear perspective of treating physicians regarding statin prescription patterns. Several misconceptions, especially regarding statin-related adverse effects, hold well among treating physicians. Insufficient implementation of dyslipidemia guidelines calls for more targeted educational interventions to achieve optimal management of patients with dyslipidemia.

## Highlights

• The STATRIP survey presents physicians' perspectives on treatment with statins.
• Misconceptions remain common, especially regarding statin-related adverse effects.
• Medical interactions, muscle aches and increase in liver enzymes are common fears.
• Educational interventions are required for optimal dyslipidemia treatment.

## 1 Introduction

Cardiovascular diseases (CVDs) remain a leading cause of morbidity and mortality worldwide. According to the World Health Organization, 32 % of all global deaths in 2019 were attributed to CVDs, of which 85 % were due to heart attack and stroke [1]. Dyslipidemia is a major modifiable risk factor contributing to the incremental burden of cardiovascular morbidity and mortality. According to most recent data from 2,078,948 participants across 133 cohorts, 39 countries, and 6 continents, presence of dyslipidemia along with other major modifiable risk factors (hypertension, underweight and overweight or obesity, diabetes, and smoking) was associated with an estimated lifetime risk of death before 90 years of age that reached 88 % in women and 94 % in men with all five risk factors, as compared to 53 % in women and 68 % in men with none of the above risk factors [2]. A strong pathophysiological background supports the targeted interventions for treatment of dyslipidemia for reducing excess cardiovascular risk. Accumulation of cholesterol-rich low density-lipoprotein (LDL) (and in particular oxidized LDL) into the vascular walls accelerates the formation and subsequent progression of the atherosclerotic lesions, subsequently resulting in clinically evident cardiovascular manifestations [3]. In line with this, substantial reductions in cardiovascular morbidity and mortality risks associated with treatment of dyslipidemia have been consistently shown beyond any doubt in large epidemiological studies. Subsequently, appropriate management of dyslipidemia is an urgent need for both primary and secondary prevention of CVDs [2].

Various potent therapeutic options for dyslipidemia are available or currently underway, including statins, ezetimibe, proprotein convertase subtilisin/kexin type 9 serine protease inhibitors (PCSK9i), newer agents like bempedoic acid, inclisiran, and potentially lipoprotein (a) [Lp(a)] inhibitors [3,4]. However, statins remain to date the primary treatment option for dyslipidemia unanimously recommended by international guidelines in the field of CVD [5,6]. Statins are in general safe and effective medications, with a small risk of side effects [3]. However, a significant portion of patients with dyslipidemia fail to achieve optimal LDL targets. In a recent study of 1909 patients with prior atherosclerotic CVD, only 41.3 % met the LDL-cholesterol target, and therapeutic inertia was acknowledged as a major contributor to suboptimal treatment [7]. Clinical inertia is interrelated with physicians’ insufficient training and education, fear of side effects, or even misjudgment of disease control [8]. Specifically regarding statins, several myths pertain among physicians and represent a clear obstacle to their implementation in clinical practice as per guidelines [5,9].

Therefore, the purpose of the present study was to report and quantify perceptions and common beliefs regarding treatment with statins, among physicians implicated in the primary and secondary care of patients with dyslipidemia. The STAtin Treatment in Routine clinical Practice (STATRIP) survey was designed with the aim of better understanding of current knowledge gaps and misperceptions regarding statin therapy, which is of paramount importance for the implementation of appropriate interventions for optimal management of dyslipidemia.

## 2 Methods

The STATRIP study was a cross-sectional questionnaire-based survey addressed to physicians prescribing statins in clinical practice. The mailing database from two scientific societies (Hellenic Society of Cardiometabolic Diseases, Obesity and Diabetes, and Greek College of General Practitioners) was used to distribute the questionnaire online. Completion of the survey was voluntary and information was provided on the objectives of the survey before participation. Participants had the opportunity to stop the survey at any stage. The study was conducted according to the principles of the Declaration of Helsinki, and approval was obtained from the Research Ethics Committee of Aristotle University of Thessaloniki (protocol number: 280/2025, date of approval: July 24, 2025).

More specifically, a structured questionnaire was developed for the purposes of this study using a freely-provided cloud-based data management tool for designing and developing web-based questionnaires. The questionnaire was distributed via e-mail to physicians. The e-mail address provided could only be used once to avoid multiple completion of the survey by the same user. Only questionnaires with full responses could be submitted. The questionnaire consisted of five parts: part I, collections of participants' demographics; part II, physicians' estimates on their common clinical practice for patients with dyslipidemia and related concerns; part III, physicians' general perceptions on usual statin prescription patterns; part IV, reported physicians' interaction with their patients regarding management of dyslipidemia, and part V, participants’ perceptions on the implementation of residual risk in clinical practice.

### 2.1 Statistical analysis

Data analysis was performed using Statistical Package for Social Sciences, SPSS Inc., Chicago, IL, USA software, version 22. Qualitative analysis variables were expressed as frequencies (percentages). Pearson chi-square test was applied for comparison of the responses provided by general practitioners and internists. A probability value of p < 0.05 was considered statistically significant.

## 3 Results

**Fig. 1:**
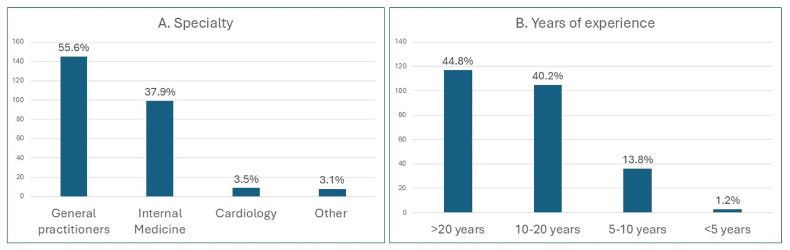
Classification of study participants according to their specialty (A) and years of experience (B).

Of the 275 physicians who were approached to participate in the study, 261 filled out the survey, which corresponds to an overall response rate of 94.9 %. As presented in Fig. 1, the majority of the responders were general practitioners (55.6 %), followed by specialists in Internal Medicine (37.9 %) (Fig. 1A). Physicians were experienced in medical practice, with more than 10 years of medical experience in their vast majority (85 %) (Fig. 1B).

**Fig. 2:**
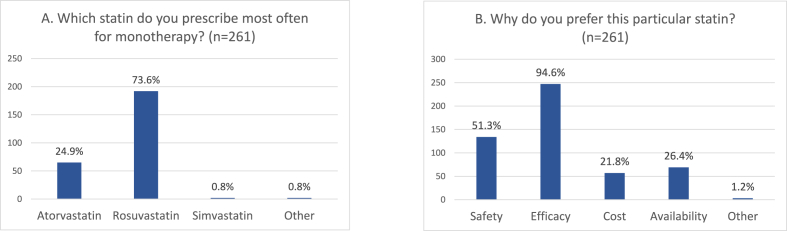
Statin of choice for monotherapy among physicians (**A**) and reasons for their choice (**B**).

Table 1 presents the survey results regarding their common clinical practice for patients with dyslipidemia and related concerns. Approximately two-thirds (67.4 %) of health care providers participating in the survey stated that they were involved in the medical care of more than 200 patients with dyslipidemia, of whom 38.3 % were classified as at least high (high or very high) cardiovascular risk. Remarkably, approximately 1 in 4 patients with dyslipidemia had bever received hypolipidemic medication before (statin-naïve). Multipharmacy was commonly reported by physicians among their elderly patients with dyslipidemia, and almost half of the study participants (49 %) reported that their elderly patients with dyslipidemia received on average 4 medications. Statin interactions with concomitant medications was a major concern, and this was more exaggerated for patients under rosuvastatin (49.8 %), and less pronounced for patients under pitavastatin (only 5.4 %). Still, rosuvastatin is the most frequently prescribed statin for monotherapy (73.6 %), for reasons related mainly to its perceived efficacy (94.6 %) (Fig. 2). Most physicians are influenced by guidelines for statin prescription, although cost plays a role in statin selection (41.0 %). Muscle aches and pain was the most commonly observed side-effect associated with statin use as reported by 85.8 %, followed by increase in liver enzymes (53.3 %) and gastrointestinal disorders (14.6 %). Interestingly, physicians reported that almost 1 in 3 of their patients who were prescribed rosuvastatin (29.2 %) complained of myalgia, 1 in 4 (26.5 %) under atorvastatin. By contrast, this complaint was very infrequent among patients receiving pitavastatin and pravastatin (7 % and 5.9 %, respectively).

**Table 1:** Physicians’ estimates on their common clinical practice for patients with dyslipidemia and related concerns.^a^

Question	All participants (n = 261)	General Practitioners^b^ (n = 145)	Internal Medicine^b^ (n = 99)
1. How many patients with dyslipidemia do you treat?			

>200	67.4 % (61.4–74)	69.0 % (60.8–76.4)	66.7 % (56.5–75.8)
100-200	17.6 % (13.2–22.3)	18.6 % (12.6–25.9)	15.2 % (8.71–23.8)
50-100	12.6 % (8.8–17.3)	11.7 % (6.9–18.1)	15.2 % (8.71–23.8)
<50	2.3 % (0.08–4.9)	0.7 % (0.02–3.8)	3.0 % (0.06–8.6)

2. How many of your patients would you classify in the following categories of cardiovascular risk?			

Very high	14.5 % (10.5–19.4)	14.4 % (9.1–21.3)	14.7 % (7.9–22.6)
High	23.8 % (18.7–29.4)	23.4 % (16.8–31.2)	24.2 % (16.1–33.9)
Moderate	32.2 % (26.5–38.2)	32.4 % (24.9–40.7)	31.6 % (23.3–42.5)
Low	30.6 % (25.1–36.6)	30.6 % (23–38.5)	31.1 % (22.3–41.4)

3. What percentage of your patients had never received hypolipidemic medication (statin-naïve)?			

	26.8 % (21.5–32.6)	26.1 % (19.2–34.1)	28.0 % (19.7–38.2)

4. How many medications on average does your >65 year old patient with dyslipidemia take?			

3	31.8 % (26.2–37.8)	28.3 % (21.1–36.3)	36.4 % (26.9–46.6)
4	49.0 % (42.8–55.3)	49.7 % (41.2–58.1)	48.5 % (38.3–58.7)
5	19.2 % (14.6–24.5)	22.1 % (15.6–29.7)	15.6 % (8.7–23.7)

5. Which statin are you most concerned about the possibility of interaction with other medications your patient is taking?			

Atorvastatin	20.7 % (15.9–26.1)	22.8 % (16.2–30.4)	20.2 % (12.8–29.4)
Pitavastatin	5.4 % (2.9–8.8)	4.8 % (1.9–9.7)	5.1 % (1.6–11.4)
Rosuvastatin	49.8 % (43.6–56.0)	47.6 % (39.2–56.0)	53.5 % (43.2–63.6)
Simvastatin	24.1 % (19.1–29.8)	24.8 % (18.0–32.7)	21.2 % (13.6–30.6)

6. Which statin do you prescribe most often for monotherapy?			

Atorvastatin	24.9 % (19.8–30.6)	22.1 % (15.6–29.7)	31.3 % (22.3–41.4)
Rosuvastatin	73.6 % (67.7–78.8)	75.9 % (68.1–82.6)	67.7 % (57.5–76.7)
Simvastatin	0.8 % (0.09–2.74)	1.4 % (0.2–4.9)	0.0 % (0.0–3.6)
Other	0.8 % (0.09–2.74)	0.7 % (0.02–3.8)	1.0 % (0.03–5.5)

7. Why do you prefer this particular statin?			

Safety	51.3 % (45.1–57.5)	53.1 % (44.6–61.4)	53.5 % (43.2–63.6)
Efficacy	94.6 % (91.1–97.0)	94.5 % (89.4–97.6)	93.9 % (87.2–97.7)
Cost	21.8 % (17–27.3)	21.4 % (15.0–28.9)	26.3 % (17.9–36.1)
Availability	26.4 % (21.2–32.2)	26.2 % (19.2–34.1)	29.3 % (20.6–39.3)
Other	1.2 % (0.2–3.3)	0.7 % (0.02–3.8)	2.0 % (0.2–7.1)

8. What factors influence your choice of which statin to prescribe?			

Heart Risk Score	72.0 % (66.1–77.4)	75.9 % (68.1–82.6)	65.7 % (55.4–74.9)
Special patient characteristics	59.4 % (53.1–65.4)	60.0 % (51.5–68.0)	58.6 % (0.09–2.74)
Guidelines	74.7 % (69.0–79.9)	77.9 % (70.3–84.4)	69.7 % (59.6–78.5)
Clinical studies	49.4 % (43.2–55.6)	46.2 % (37.9–54.7)	51.5 % (41.2–61.7)

9. How important do you consider the cost in statin selection?			

Not at all important	1.9 % (0.06–4.4)	0.0 % (0.0–2.5)	3.0 % (0.06–8.6)
Somewhat important	7.3 % (4.4–11.1)	6.9 % (3.3–12.3)	8.1 % (3.5–15.3)
Important	41.0 % (35.0–47.2)	40.0 % (32.0–48.5)	47.5 % (37.3–57.8)
Very important	15.7 % (11.5–20.7)	20.0 % (13.8–27.4)	9.1 % (4.2–16.5)

6. What are the most common side-effects you have observed in patients taking statins?			

Increase in liver enzymes	53.3 % (47.0–59.4)	55.9 % (47.4–64.1)	51.5 % (41.2–61.7)
Gastrointestinal disorders	14.6 % (10.5–19.4)	15.9 % (10.3–22.8)	12.1 % (6.4–20.2)
Muscle aches and pains	85.8 % (80.1–89.8)	84.1 % (77.1–89.7)	87.9 % (79.8–93.6)
Others	1.9 % (0.06–4.4)	2.8 % (0.07–6.9)	0.0 % (3.6)

7. What percentage of your patients given the following statin has complained of myalgia?			

Simvastatin	16.1 % (11.8–21.1)	16.5 % (10.9–23.6)	15.2 % (8.7–23.7)
Atorvastatin	26.4 % (21.2–32.2)	26.2 % (19.2–34.1)	26.0 % (17.1–35.0)
Rosuvastatin	29.1 % (23.7–35.0)	28.3 % (21.1–36.3)	30.7 % (21.5–40.3)
Pravastatin	5.9 % (3.2–9.3)	5.5 % (2.4–10.6)	6.1 % (2.2–12.7)
Pitavastatin	7.0 % (4.1–10.7)	6.2 % (2.9–11.4)	8.1 % (3.5–15.3)

a Results are expressed as % (95 % Confidence Intervals).

b P values were non-significant for all comparisons of responses between General Practitioners and Internal Medicine physicians.

Table 2 presents physicians’ general perceptions on usual statin prescription patterns. All participants stated that statin monotherapy is at least moderately effective in lowering cholesterol. However, only 10.3 % of the statin participants were confident that at least 80 % of their patients were adequately treated with respect to target LDL values according to their cardiovascular risk category. Addition of ezetimibe without dose titration is the most common strategy followed by participants if the LDL target is not met (53.6 %), and it is more frequently combined with either rosuvastatin (37.4 %) or atorvastatin (33.0 %). Only 23 % would proceed with dose titration, and an even lower portion (16.9 %) would proceed with transition to a high dose statin. Main concerns reported on statin monotherapy include insufficient cholesterol lowering (62.8 %), adverse reactions (59.8 %), and interactions with other medications (23.8). Statin monotherapy was reported as the treatment of choice in patients with low cardiovascular risk (82.4 %).

**Table 2:** Physicians’ general perceptions on usual statin prescription patterns.^a^

Question	All participants (n = 261)	General Practitioners^b^ (n = 145)	Internal Medicine^b^ (n = 99)
1. What is your opinion on the effectiveness of statin monotherapy in lowering cholesterol?			

Very effective	64.0 % (57.8–69.8)	64.8 % (56.5–72.6)	64.7 % (54.4–74.0)
Quite effective	15.7 % (11.5–20.7)	13.1 % (8.08–19.7)	19.2 % (12.0–28.3)
Moderately effective	20.3 % (15.6–25.7)	22.1 % (15.6–29.7)	16.2 % (9.5–24.9)

2. What percentage of your patients do you consider to be adequately treated with respect to target LDL values according to their cardiovascular risk category?			

80 %–100 %	10.3 % (6.9–14.7)	11.7 % (7.0–18.1)	8.1 % (3.6–15.3)
60 %–79 %	50.6 % (44.3–56.8)	50.3 % (41.9–58.8)	49.5 % (39.3–59.7)
40 %–59 %	31.0 % (25.5–37.0)	30.3 % (23.0–38.5)	33.3 % (24.2–43.5)
20 %–39 %	8.1 % (5.1–12.0)	7.6 % (3.9–13.2)	9.1 % (4.2–16.6)

3. If the LDL target is not met, in primary prevention, which of the following do you apply?			

Addition of ezetimibe without dose titration	53.6 % (47.4–59.8)	54.5 % (46.0–62.8)	50.5 % (40.3–60.7)
Dose titration	23.0 % (18.0–28.6)	22.8 % (16.2–30.5)	22.2 % (14.5–31.7)
Administration of medium strength statin	5.4 % (3.0–8.8)	6.9 % (3.4–12.3)	4.0 % (1.1–10.0)
Administration of high strength statin	16.9 % (12.5–22.0)	15.2 % (9.8–22.0)	21.2 % (13.6–30.6)
Other	1.2 % (0.2–3.3)	0.7 % (0.0–3.8)	2.0 % (0.3–7.1)

4. Which statin is ezetimibe more frequently combined with in your patients?			

Simvastatin	19.3 % (14.6–24.5)	19.8 % (13.8–27.4)	18.9 % (12.0–28.3)
Atorvastatin	33.0 % (27.3–39.0)	31.9 % (24.3–40.0)	34.1 % (25.1–44.6)
Rosuvastatin	37.4 % (31.7–43.7)	35.7 % (28.1–44.2)	39.1 % (29.7–49.7)

5. How do you compare statin monotherapy with other treatment options for dyslipidemia in primary prevention?			

It is superior	46.0 % (39.8–52.2)	42.1 % (33.9–50.5)	53.5 % (43.2–63.6)
It is equivalent	22.6 % (17.7–28.1)	21.4 % (15.0–29.0)	24.2 % (16.2–33.9)
It is inferior	28.4 % (23.0–34.2)	31.0 % (23.6–39.2)	22.2 % (14.5–31.7)
I have no opinion	3.1 % (1.3–6.0)	5.5 % (2.4–10.6)	0.0 % (0.0–3.7)

6. What are your main concerns about statin monotherapy?			

Interactions with other medications	23.8 % (18.7–29.4)	25.5 % (18.7–33.4)	20.2 % (12.8–29.5)
Insufficient cholesterol lowering	62.8 % (56.7–68.7)	62.1 % (53.7–70.0)	62.6 % (52.3–72.2)
Adverse reactions	59.8 % (53.6–65.8)	54.5 % (46.0–62.8)	63.6 % (53.4–73.1)
Long-term use	13.0 % (9.2–17.7)	13.8 % (8.6–20.5)	11.1 % (5.7–19.0)
Others	1.5 % (0.4–3.9)	1.4 % (0.2–4.9)	2.0 % (0.3–7.1)

7. Are there specific types of patients for whom you prefer statin monotherapy?			

Patients at very high risk of cardiovascular events	6.5 % (3.8–10.2)	6.9 % (3.4–12.3)	6.1 % (2.3–12.7)
Patients at risk of cardiovascular events	10.3 % (6.9–14.7)	9.7 % (5.4–15.7)	11.1 % (5.7–19.0)
Patients at moderate risk of cardiovascular events	47.9 % (41.7–54.1)	44.1 % (35.9–52.6)	51.5 % (41.3–61.7)
Patients at low risk of cardiovascular events	82.4 % (77.2–86.8)	86.2 % (79.5–91.4)	77.8 % (68.3–85.5)

8. What percentage of your treated dyslipidemic patients receive a combination of ezetimibe-statin?			

0–25 %	19.2 % (14.6–24.5)	20.0 % (13.8–27.4)	20.2 % (12.8–29.5)
26–50 %	56.7 % (50.5–62.8)	56.6 % (48.1–64.8)	55.6 % (45.2–65.6)
51–75 %	22.6 % (17.7–28.2)	22.1 % (15.6–29.7)	22.2 % (14.5–31.7)
76–100 %	1.5 % (0.4–3.9)	1.4 % (0.2–4.9)	2.0 % (0.3–7.1)

a Results are expressed as % (95 % Confidence Intervals).

b P values were non-significant for all comparisons of responses between General Practitioners and Internal Medicine physicians.

Patient-physician interaction upon statin prescription is reported in Table 3. Patient compliance is admittedly very important (77.4 %). The majority of physicians (77.1 %) spend more than 10 min to educate their patients, and approximately half of physicians (52.5 %) provide written instructions on statin therapy. Adverse reactions (88.5 %) and duration of treatment (81.2 %) are the most common patients’ concerns, which are most frequently addressed by physicians by providing further information (92.7 %) and encouraging communication (65.5 %).

**Table 3:** Reported physicians’ interaction with their patients regarding management of dyslipidemia.^a^

Question	All participants (n = 261)	General Practitioners^b^ (n = 145)	Internal Medicine^b^ (n = 99)
1. How important do you consider patient compliance with statin therapy?			

Very important	77.4 % (71.8–82.3)	75.9 % (68.1–82.6)	81.8 % (72.8–88.9)
Moderately important	22.2 % (17.3–27.8)	23.5 % (16.8–31.2)	18.2 % (11.2–27.2)
Important	0.4 % (0.0–2.1)	0.7 % (0.0–3.8)	0.0 % (0.0–3.7)

2. How much time do you spend educating your patients about treatment for dyslipidemia?			

More than 15 min	44.4 % (38.3–50.7)	46.9 % (38.6–55.4)	41.4 % (31.6–51.8)
10–15 min	32.6 % (26.9–38.6)	27.6 % (20.5–35.6)	37.4 % (27.9–47.7)
5–9 min	11.5 % (7.9–16.0)	14.5 % (9.2–21.3)	8.1 % (3.6–15.3)
Less than 5 min	11.5 % (7.9–16.0)	11.0 % (6.4–17.3)	13.1 % (7.2–21.4)

3. Do you provide your patients with written information about statin therapy?			

Yes	52.5 % (46.2–58.7)	58.6 % (50.2–66.7)	47.5 % (37.3–57.8)
No	47.5 % (41.3–53.8)	41.4 % (33.3–49.9)	52.5 % (42.2–62.7)

4. What are the most common questions patients ask you about statins?			

Adverse reactions	88.5 % (84.0–92.1)	84.8 % (77.9–90.2)	93.9 % (87.3–97.7)
Duration of treatment	81.2 % (76.0–85.8)	82.8 % (75.6–88.5)	82.8 % (73.9–89.7)
Administration mode	44.1 % (37.9–50.3)	42.1 % (33.9–50.5)	45.5 % (35.4–55.8)

5. How do you address your patients' concerns about the side effects of statins?			

Encourage communication	65.5 % (59.4–71.3)	65.5 % (57.2–73.2)	64.7 % (54.4–74.0)
Provide information	92.7 % (88.9–95.6)	95.9 % (91.2–98.5)	89.9 % (82.2–95.1)
Discuss alternatives	26.4 % (21.2–32.2)	30.3 % (23.0–38.5)	19.2 % (12.0–28.3)
Others	0.4 % (0.0–2.1)	0.7 % (0.0–3.8)	0.0 % (0.0–3.7)

a Results are expressed as % (95 % Confidence Intervals).

b P values were non-significant for all comparisons of responses between General Practitioners and Internal Medicine physicians.

Finally, the study reported on participants’ perceptions on the implementation of residual risk in clinical practice, which are presented in detail in Supplementary Table 1. Physicians consider themselves informed in latest research on residual cardiovascular risk factors (95.0 %). Most of them have attended conferences or seminars on the role of non-HDL & Lp(a) (76.3 %) and are positive that these factors will influence their practices regarding statin prescription in the future (82.4 %). As many as 85.4 % take into account non-HDL and Lp(a) values for the calculation of the 10-year cardiovascular risk (Heart Risk Score), although only 58.6 % stated that they use non-HDL cholesterol measurements in their daily clinical practice. Only 41 % of study participants have ever asked their patients for an ApoB measurement.

## 4 Discussion

The STATRIP survey provides information regarding common perceptions, knowledge and beliefs regarding treatment with statins, obtained from a considerable population of physicians primarily involved in the primary and secondary care of patients with dyslipidemia. Remarkably, all results were homogeneously distributed across general practitioners and internists. Several findings of the present study merit further attention.

First, study participants have clearly expressed their concerns regarding statin-related side effects. These include fears on interactions with other medication, muscle aches and pain, increase in liver enzymes, and gastrointestinal disorders. These concerns are more exaggerated with rosuvastatin, and far less pronounced with pitavastatin or pravastatin. When physicians were asked about the frequency of these side-effects, they reported that almost 1 in 3 of their patients who were prescribed rosuvastatin complained of myalgia, and 1 in 4 under atorvastatin (Table 1). These results overtly demonstrate that perceived side-effects of statins remain remarkably overestimated among treating physicians. Statin-associated muscle symptoms cover a broader range of clinical presentations, usually with normal or minimally elevated serum creatine kinase (CK) levels, and are indeed the predominant adverse effects encountered in clinical practice. Although a varying prevalence of 7–29 % has been reported in registries and observational studies, this percentage drops to as low as 2 % when it comes to randomized controlled trials (RCTs) [10,11]. Statin-associated myopathy, with significant elevation of CK levels, is a serious but admittedly very rare side effect of statins, affecting 1 per 1000 to 1 per 10,000 people on standard statin doses [10]. The so-called nocebo effect, i.e., excess rate of muscle-related adverse events reported only when patients and their doctors were aware of statin use and not when its use was blinded, has been illustrated as the reason for this pronounced discrepancy [12]. Furthermore, statins are associated with transient increases in liver enzymes in only 0.5–2 % of patients treated with statins, but are not clinically relevant; idiosyncratic liver injury attributed to statin use is very rare, and causality is difficult to prove [11].

Second, although all participants consider statins to be effective hypolipidemic drugs, 9 in 10 physicians admit that their patients are not adequately controlled with respect to target LDL values (Table 2). Suboptimal management of patients with dyslipidemia with respect to their LDL cholesterol values is a major global problem substantially contributing to the increased burden of CVDs. Rather disappointingly, suboptimal control rates of dyslipidemia remain remarkably stable over time. According to recently published data from NHANES, among US adults receiving statin therapy, age-adjusted lipid control did not significantly change from 78.5 % in 2007–2008 to 79.5 % in 2017–2018 [13]. A well-acknowledged strategy of improving LDL cholesterol values is combination lipid-lowering therapy. Data from tenths of thousands of individuals demonstrate that compared with statin monotherapy, combination lipid-lowering therapy with statins and ezetimibe is associated with an overall higher reduction in LDL, the same risk of adverse effects, and significantly lower risk of all-cause mortality, major adverse cardiovascular events, and stroke [14]. Nevertheless, almost half of the responders consider statin monotherapy to be superior to other treatment options for dyslipidemia, and only 53.6 % would consider addition of ezetimibe if the LDL target is not met. Accordingly, 75.9 % of responding physicians reported that less than half of their patients receive a combination of ezetimibe-statin. These data underscore the importance of educating physicians further to enable the incorporation into routine clinical practice of combination lipid-lowering therapy for uncontrolled patients with dyslipidemia.

Third, physicians participating in the survey appear to be aware of the significance of patients' involvement in therapeutic decisions, and willing to address their patients’ concerns (Table 3). In a recent nationwide cohort study of 151,791 apparently healthy Danish adults aged 40–85 with no prior cardiovascular disease or major comorbidities, high adherence to statins over five years was associated with significantly lower absolute risk of major adverse cardiovascular events (3.01 % versus 4.77 % for low adherence) and a substantial 34 % relative risk reduction, with consistent benefits across sexes and age groups [15]. This study emphasizes the need for personalized approaches when making therapeutic decisions that enhance adherence in order to achieve cardiovascular benefits.

Finally, although responders consider themselves informed on the implementation of residual cardiovascular risk and recognize its significance in optimal therapeutic strategies, only 58.6 % apply non-HDL cholesterol measurements in their clinical practice (Supplementary Table). Recently, many efforts have been directed at elucidating the role of low high-density-lipoprotein cholesterol, high triglycerides, especially triglyceride-rich lipoproteins, and lipoprotein (a) in the atherosclerotic plaque formation and progression of atherosclerotic cardiovascular disease [16]. While new and hopeful treatments targeting residual cardiovascular risk are under way, optimizing current therapeutic options, improving adherence through patient-doctor interaction, and combating physicians’ inertia remain the cornerstone implementation strategies for treatment of dyslipidemia in the context of optimal cardiovascular risk management.

Few studies have previously attempted to quantify physicians' perceptions and knowledge regarding routine clinical practice for management of dyslipidemia. In a heterogeneous sample of 496 responding pharmacists from governmental and private hospitals and those working in private clinics or community pharmacies, it was reported that physicians and pharmacists had suboptimal clinical knowledge regarding statin therapy, dose intensities, contraindications, and monitoring parameters, while the lowest knowledge were related to statin-drug interactions [17]. Another survey of 260 doctors working in primary care, residents and family medicine specialists revealed many misconceptions among patient about the side effects of statins often leading to refusals to initiate or discontinuation of therapy, but doctors were interviewed to respond to their patients’, and not their own, perspectives with regard to treatment of dyslipidemia [18].

Strengths of the STATRIP study include a considerable number of responding physicians and a homogeneous population of doctors primarily implicated in the therapeutic management of patients with dyslipidemia. The high response rate of the present study further strengthens the impact of the study findings. A wide range of issues involved in statin prescription patterns was addressed. Limitations of the study include the subjective estimate of the responders, which is an inherent limitation of the study. Respondents might be more interested or better informed about dyslipidemia management, resulting in potential self-selection bias and reducing generalizability. Responders were sought from a single country, which may limit generalizability of the study findings. Finally, despite the high response rate, it could be speculated that physicians registered in the Hellenic Society of Cardiometabolic Diseases, Obesity and Diabetes, and the Greek College of General Practitioners, may be more motivated as compared to the general population of medical doctors implicated in the care of patients with dyslipidemia.

The present study has direct clinical implications. Clinical inertia and deviations from optimal management of patients with dyslipidemia are clearly reflected in the study. These findings call for targeted educational interventions to facilitate the implementation of residual risk in clinical practice and familiarize physicians with cornerstone implementation strategies for treatment of dyslipidemia.

In conclusion, the STATRIP survey provides a clear perspective of physicians primarily implicated in the management of patients with dyslipidemia, regarding treatment with statins and relevant prescription patterns. Several misconceptions, especially regarding statin-related adverse effects, hold well among treating physicians. Insufficient implementation of dyslipidemia guidelines is an obvious barrier to optimal management of patients with dyslipidemia, and further highlights the need for physician-targeted educational interventions.

## Financial disclosure

The study was supported by a grant from ELPEN Pharmaceutical Company, 10.13039/100014283Hellenic Society of Cardiometabolic Diseases, Obesity and Diabetes, and Greek College of General Practitioners.

## Declaration of competing interest

The authors declare the following financial interests/personal relationships which may be considered as potential competing interests: Vasilios Kotsis reports financial support was provided by ELPEN Pharmaceutical Company. Vasilios Kotsis reports financial support was provided by Hellenic Society of Cardiometabolic Diseases, Obesity and Diabetes. Vasilios Kotsis reports financial support was provided by Greek College of General Practitioners. If there are other authors, they declare that they have no known competing financial interests or personal relationships that could have appeared to influence the work reported in this paper.
