# Supplementary material for: Statin treatment in routine clinical practice: Insights from the STATRIP physician survey
Source: Atheroscler Plus. 2026 Jan 17;63:28–33. doi: 10.1016/j.athplu.2026.01.003 (PMC12857172; doi:10.1016/j.athplu.2026.01.003)
Supplement: Multimedia component 1 [file mmc1.docx]

| **Supplementary table 1.** Participants’ perceptions on the implementation of residual risk in clinical practice.* | | | |
| --- | --- | --- | --- |
| **Question** | **All participants**  **(n=261)** | **General Practitioners****  **(n=145)** | **Internal Medicine****  **(n=99)** |
| 1. Are you aware of the latest developments in research on residual cardiovascular risk factors? | | | |
| Yes  No | 95.0% (91.6-97.3)  5.0% (2.7-8.4) | 94.5% (89.4-97.6)  5.5% (2.4-10.6) | 95.0% (88.6-98.3)  5.1% (1.7-11.4) |
| 2. Have you recently attended conferences or seminars on the role of non-HDL & Lp(a) in residual cardiovascular risk? | | | |
| Yes  No | 76.3% (70.6-81.3)  23.8% (18.7-29.4) | 79.3% (71.8-85.6)  20.7% (14.4-28.2) | 70.7% (60.7-79.4)  29.3% (20.6-39.3) |
| 3. Do you think these factors will influence your practices regarding statin prescription in the future? | | | |
| Yes  No  Don't know | 82.4% (77.2-86.8)  8.1% (5.1-12.0)  9.6% (6.3-13.8) | 83.5% (76.4-89.1)  6.2% (2.9-11.5)  10.3% (5.9-16.5) | 82.8% (73.9-89.7)  9.1% (4.2-16.6)  8.1% (3.6-15.3) |
| 4. Do you use non-HDL cholesterol (= total cholesterol minus HDL cholesterol) measurement in your daily clinical practice? | | | |
| Yes  No | 58.6% (52.4-64.7)  41.4% (35.3-47.6) | 61.4% (52.9-69.3)  38.6% (30.7-47.1) | 55.6% (45.2-65.6)  44.4% (34.5-54.8) |
| 5. Do you take into account non-HDL & Lp(a) values for the calculation of the 10-year cardiovascular risk (Heart Risk Score)? | | | |
| Yes  No  Other | 85.4% (80.6-89.5)  14.6% (10.5-19.4)  0.4% (0.0-2.1) | 83.5% (76.4-89.1)  16.6% (10.9-23.6)  0.0% (0.0-2.5) | 87.9% (79.8-93.6)  12.1% (6.4-20.2)  1.0% (0.0-5.5) |
| 6. Would you use in your daily clinical practice, a 10-year cardiovascular risk calculator app (Heart Risk Score) that would calculate risk taking into account non-HDL & Lp(a)? | | | |
| Yes  No  Maybe | 85.8% (80.1-89.8)  5.8% (3.3-9.3)  8.4% (5.4-12.5) | 86.2% (79.5-91.4)  4.1% (1.5-8.8)  9.7% (5.4-15.7) | 86.9% (78.6-92.8)  6.1% (2.3-12.7)  7.1% (2.9-14.0) |
| 7. Have you ever asked your patient for an ApoB measurement? | | | |
| Yes  No | 41.0% (35.0-47.2)  59.0% (52.8-65.0) | 35.9% (28.1-44.2)  64.1% (55.8-71.3) | 47.5% (37.3-57.8)  52.5% (42.2-62.7) |
| 8. Do you agree with the new LDL-C targets as set out in the recent 2023 Hellenic Atherosclerosis Society Guidelines for the Diagnosis and Treatment  of Dyslipidemias? | | | |
| I totally agree  I have doubts but I will apply them  I will not apply them at all | 64.4% (58.2-70.2)  34.9% (29.0-40.1)  0.8% (0.1-2.7) | 60.7% (52.2-68.7)  38.6% (30.7-47.1)  0.7% (0.0-3.8) | 68.7% (58.6-77.6)  30.3% (21.5-40.4)  1.0% (0.0-5.5) |
| *Results are expressed as % (95% Confidence Intervals).  **P values were non-significant for all comparisons of responses between General Practitioners and Internal Medicine physicians. | | | |
